# Common carotid artery puncture in anterior circulation thrombectomy in patients with unfavorable vascular anatomy: A case-control study

**DOI:** 10.1097/MD.0000000000017477

**Published:** 2019-10-04

**Authors:** Zhengzhou Yuan, Jinglun Li, Muke Zhou, Hongbo Zheng, Hua Luo, Xiu Chen, Zuoxiao Li, Li He

**Affiliations:** a West China Hospital of Sichuan University, Department of Neurology, ChenDu; b Affiliated Hospital of Southwest Medical University, Department of Neurology, LuZhou, China.

**Keywords:** acute ischemic stroke, carotid artery puncture, large vessel occlusion, mechanical thrombectomy, unfavorable vascular anatomy

## Abstract

The objective of this study was to compare clinical outcomes in patients who with unfavorable vascular anatomy underwent mechanical thrombectomy (MT) by common carotid artery access versus transfemoral approach.

A retrospective review was performed in our hospital database to identify patients with challenging vascular anatomy who underwent MT for anterior circulation large vessel occlusion (LVO) between August 2015 and November 2018. Transcarotid and transfemoral cohorts were compared. Patient characteristics, procedural techniques, clinical outcomes were recorded.

A total of 52 patients were included, 16 (31%) underwent MT via transcarotid access. There were no significant differences in patient characteristics, intravenously recombinant tissue plasminogen activator therapy, clot location, or carotid tortuosity and presence of aortic arch type. There were significant differences in clinical outcomes between the 2 cohorts, including mean access-to-reperfusion time (84 vs 44 minutes; *P* = .000), poor clinical outcome (modified Rankin scale >2) at 90 days follow-up (37.5% vs 63.9%; *P* = .034). But there were no significant differences in successful revascularization rates (thrombolysis in cerebral infarction score ≥2b 87.5% vs 80.6%; *P* = .541), post-thrombectomy symptomatic intracranial hemorrhage (12.5% vs 13.9%; *P* = .892), and mortality (12.5% vs 22.2%; *P* = .412) were similar between transcarotid and transfemoral cohorts.

Our results demonstrate that transcarotid access for MT of anterior circulation LVO in patients with unfavorable vascular anatomy may be considerable. Transcarotid access may be better than transfemoral access in well-selected unfavorable vascular anatomy patients undergoing MT.

## 1 Introduction

Seven prospective randomized trials showed that mechanical thrombectomy (MT) was superior to standard medical care in patients with acute ischemic stroke (AIS) caused by anterior circulation large vessel occlusion (LVO).^[1–7]^ Further analysis of these studies showed that there was a strong association between longer revascularization times and the poor function outcome.^[8]^ Therefore, shorter procedure times would have a positive effect on patients with AIS undergoing MT. However, unfavorable vascular anatomy, such as severe tortuosity aortic arch, kinking and coiling internal carotid artery (ICA) present technical challenges to performing MT efficiently, ultimately leading to significantly delay or even preclude recanalization.^[9]^

For carotid artery stenting or aneurysm coil embolization procedures in which the standard percutaneous transfemoral access cannot be established due to unfavorable vascular anatomy, alternative approaches such as direct transcarotid access can be used.^[10,11]^ And a few cases of direct common carotid puncture for endovascular thrombectomy have been reported.^[12,13]^ Here we present our experience in AIS patients with unfavorable vascular anatomy who underwent MT for anterior circulation LVO via direct common carotid artery puncture versus transfemoral approach.

## 2 Methods

### 2.1 Patient selection and evaluation

We retrospectively studied a database of all patients with AIS with unfavorable vascular anatomy who had undergone MT by 2 primary operators at the Affiliated Hospital of Southwest Medical University, Luzhou, Sichuan, China from August 2015 to November 2018. The study was approved by the institutional review board (No. 201901035). All cohort patients underwent computed tomography angiography (CTA) of the head and neck, including the aortic arch, the unfavorable vascular anatomy was defined as bovine, aortic arch, dolichoarteriopathy (BAD) scores ≥2.^[9]^ BAD vessels score is according to the aforementioned anatomic criteria, bovine arch, aortic arch type, and dolichoarteriopathy of ICA. Patients with a score of 0 or 1 considered good and a score of 2 or 3 considered poor. The following patients were excluded from analysis: patients with tandem occlusion of ICA, posterior circulation LVO, patients in whom both the ICA dolichoarteriopathy and aortic arch type could not be assessed with preoperative CTA, and patients who underwent access site crossover during thrombectomy (femoral to common carotid in 2 patients). Tandem occlusion of the ICA was excluded, as the treatments are usually complex and usually requires an increase in the length of procedural, which may introduce confounding. Moreover, tandem occlusions AIS prevent adequate visualization and evaluation ICA dolichoarteriopathy on preprocedural CTA.

### 2.2 Procedure of MT

#### 2.2.1 Common carotid artery access

Direct common carotid artery access was obtained with the patients under general anesthesia or with conscious sedation. The target puncture site was 2 to 3 cm above the clavicle, the entry site is infiltrated with 2 to 3 mL of local anesthetic (2% lidocaine). The common carotid artery was punctured at 45-degree angle with 18 gauge micropuncture needle. A 0.035-inch soft tip guidewire is advanced into the ICA under X-ray fluoroscopic. Withdrawal the puncture needle and 6F sheath is placed into the ICA through 0.035-inch guidewire.^[14]^ Once 6 F sheath access is established, systemic heparin is administered to achieve a therapeutic activated coagulation time between 250 and 300 seconds, and 058 Navien (ev3-Covidien, Irvine, CA) is delivered into the distal ICA. From this point, various endovascular approaches, including the use of solitaire FR (ev3-Covidien), direct aspiration (a direct aspiration first pass technique for the endovascular treatment of stroke), based on local anatomic considerations, and location of the thrombus. We used ExoSeal vascular closure device (Cordis, Dublin, Ohio) to achieve hemostasis after sheath removal at the end of the procedure. Carotid vascular ultrasound and chest computed tomography (CT) were performed 24 to 48 hours after operation to confirm whether there were dissection, pseudoaneurysm, and pneumothorax.

#### 2.2.2 Transfemoral access

An 8 F short guide sheath is placed in the femoral artery. A coaxial system consisting of a 125 cm diagnostic catheter (ev3-Covidien) and a 260 cm 0.035-inch guidewire is advanced into the target carotid artery. The diagnostic catheter is removed and insertion Navien catheter to the end of ICA, then, a microcatheter with a microwire is introduced through the Navien catheter and navigated across the thrombus. In some cases, Simon catheter and 0.035-inch guidewire were also used to deliver Simon catheter into the target carotid artery, remove loach guidewire, exchange supporting guide wire, withdraw Simon catheter and then transfer 8F guide tube and Navein catheter to the end of the target ICA through supporting guidewire. The stent retriever device is deployed in a standard fashion and withdrawn after 5 min.

### 2.3 Study outcomes

The primary outcome was the modified Rankin scale (mRS) score (a 7-point scale ranging from 0 “no symptoms” to 6 “dead”) at 90 days post-intervention. The mRS score was evaluated at 90 days after the stroke over the telephone or outpatient follow-up. Second outcomes were single-pass recanalization rate, successful revascularization rates (modified thrombolysis in cerebral ischemia [mTICI] score ≥2b), symptomatic intracranial hemorrhage (ICH), 90-day mortality. Symptomatic ICH was defined as type 2 parenchymal hematoma with an associated National Institutes of Health Stroke Scale (NIHSS) score worsening of 4 or more points (safe implementation of thrombolysis in stroke-monitoring study classification). Type 2 parenchymal hematoma is larger than 30% of the infarcted area with an associated mass effect. And procedure-related complications were recorded. Poor clinical outcome was defined as mRS >2 point. Reperfusion was graded according to the mTICI scale score (range 0: no flow beyond the occlusion, range 1: minimal reperfusion, range 2a: less than 50% of the affect edvascular territory reperfused, range 2b: greater than 50% reperfusion, and range 3: complete reperfusion). Successful reperfusion was defined as mTICI 2b or 3.

### 2.4 Statistical analysis

All statistical tests were carried out with SPSS version 22.0.0 software (IBM, Armonk, NY). Continuous variables are expressed as means and standard deviation, and as frequency for categorical variables. Analysis was carried out using *χ*^2^, unpaired *t* test, Fisher exact tests, and Levene test for detection of significant difference between carotid and femoral access points. Univariate analysis was subsequently performed to identify potential factors associated with poor clinical outcome (mRS 3–6 at 90 days) after endovascular treatment. Statistical significance was set as *P* < .05.

## 3 Results

### 3.1 Patients and characteristics

A total of 52 AIS patients with a mean age of 76.8 years were analyzed, all patients with a BAD vessel score ≥2 underwent MT for anterior circulation LVO. The majority of patients were male 63.5%. The average NIHSS score on presentation was 14.3 (range 7–25). The M1 segment of the middle cerebral artery was the most common site of occlusion (65.3%), followed by M2 (19.2%) and then ICA terminus (15.4%). No significant differences were seen between the common carotid artery and femoral cohort as to age, sex, past history, medication, NIHSS score on admission, Alberta stroke program early CT score, intravenously recombinant tissue plasminogen activator, location of LVO, or anatomical component comprising the BAD vessel score (Table 1).

**Table 1:**
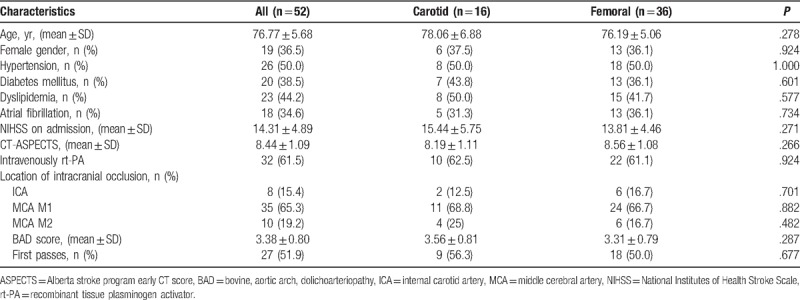
Demographics and clinical characteristics.

### 3.2 Clinical outcomes

Significant differences in technical or clinical outcomes were found between 2 access technique cohorts. The mean time from neck puncture to reperfusion was shorter than groin puncture (43.9 vs 84.3 minutes; *P* = .000). There was also significant difference in poor clinical outcome (mRS >2) at 90 days follow-up (37.5% vs 63.9%; *P* = .034) (Table 2). A mTICI score of ≥2b was considered a favorable radiographic outcome after MT. However, there were no differences in successful revascularization between transcarotid and transfemoral patients (87.5% vs 80.6%; *P* = .541), and intra-arterial recanalization was achieved after a single pass of the stent retriever in 56.3% of transcarotid cases and 50.0% of transfemoral cases (*P* = .677). There were also no differences in post-thrombectomy symptomatic ICH. Mortality rate at 90 days was not significantly different in the transcarotid group than the transfemoral group (12.5% vs 22.2%; *P* = .412) (Table 2). As for puncture complications, no pneumothorax or common carotid artery dissection was found in carotid puncture patients. There was 1 pseudoaneurysm in transcarotid and transfemoral patients, which disappeared after compression.

**Table 2:**
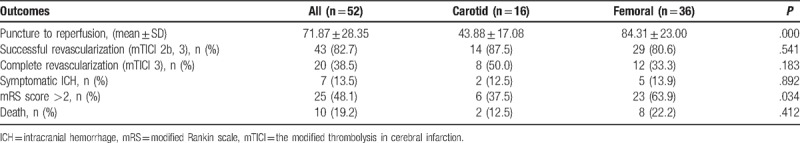
Summary of technical and clinical outcomes.

## 4 Discussion

Carotid artery puncture has been adopted for cerebral angiography more than half a century. Since 1960s,^[15]^ the initial cerebral angiography procedures were performed through direct carotid artery puncture or transbrachial access. As the technology developed further and transfemoral catheters were introduced, the transfemoral approach became the standard technique in establishing access during neuroendovascular interventions, including cerebral angiography and stroke interventions. Transradial or transbrachial approach has been utilized in CAS and may be considered as another alternative access to stroke interventions, especially in the cases with unfavorable vascular anatomy, such as bovine origin left carotid artery or tortuous right carotid artery, where a challenging transfemoral route is anticipated. But even with these techniques, there still exists a difficult access cases, such access technical is difficult for catheterization of a nonbovine with sharp angle left ICA take-off.^[16]^

Previous studies have shown that complex aortic arch and carotid artery anatomy are associated with increased technical difficulty,^[9]^ thus prolonging the recanalization time and increasing the incidence of complications and poor function outcome.^[17]^ Such as Vitek or Simmons type inner catheters, can enter the carotid artery through the femoral artery approach, and the radial artery approach also provides a more advantageous path for patients with type II or III aortic arch or bovine configuration of the left common carotid artery to quickly enter the intracranial artery, but for an acute angle between the innominate and left common carotid artery is more challenging for catheter and guidewire into the ICA via transradial access.^[18]^

Direct carotid artery puncture may be an alternative method for patient with unfavorable vascular anatomy. As far as we know, there only a few case reports which adopted transcarotid artery approach for AIS thrombectomy.^[12,13]^ These cases introduced direct carotid artery puncture is a feasible alternative to an anatomically difficult transfemoral access; however, these reports are cases report, we performed a quantitative analysis of this method in our case-control sturdy. The most common problem of carotid artery puncture is the fear of puncture complications, especially arterial dissection and hematoma formation at the closure of the puncture site.^[14]^ Because puncture site subcutaneous hematoma formation may causes lethal tracheal obstruction, and occlusion of the puncture artery can also end up with ischemic stroke. The use of vascular closure devices greatly reduces the incidence of hematoma and makes carotid puncture safer. By using vascular closure devices, we did not find carotid dissection or hematoma. The common carotid artery puncture bypasses the tortuous aortic arch, and the catheter guide wire travels shorter in vivo, which is more controllable.^[13]^ Therefore, we hypothesized that a common carotid artery puncture could provide benefit in successful and efficient reperfusion compared with the standard transfemoral access.

Our results are noteworthy for several reasons. First, just as allied forces bypass the Maginot Line, common carotid artery puncture directly bypasses the bad aortic arch or the tortuous abdominal aorta or radial artery and quickly and easily enters the target side of the carotid artery. Second, significant difference was seen in technical outcome or efficacy between transcarotid and transfemoral groups. The recanalization time, and mRS at 3 months follow-up were better in transcarotid group than in transfemoral group. Significant benefit of the transcarotid approach over the standard transfemoral group, particularly if this finding can be replicated in larger multicenter studies across unfavorable vascular anatomy. Third, all operators in this series were certainly more experienced and technically adept at thrombectomy via transfemoral access, but there is a considerable learning curve associated with performing the transcarotid access. With further improvement and optimization, the potential for improving efficiency and efficacy of thrombectomy via transcarotid is more significant. Moreover, access catheters and guide systems have been designed for transfemoral primarily and there are no commercially available systems designed specifically for transcarotid. With the development of newer devices and technology, there is great potential for taking better advantage of the anatomical configuration of the great vessels approached via common carotid artery. We encourage neurointerventionists to consider direct carotid puncture as an alternative to traditional femoral artery interventions for AIS patients with challenging anatomy.

## 5 Limitations

This study has several limitations, including our study design is a retrospective case-control study with no randomized grouping of cases. To our knowledge, this is the first and largest reported series comparing transcarotid and transfemoral for anterior circulation thrombectomy, but it only included a small number of patients with unfavorable vascular anatomy who underwent either common carotid or femoral thrombectomy. Second, severe complications, such as cervical hematoma and dissection, have been reported in the past. Some of these complications are catastrophic. With the development of new materials, this risk has been reduced, but it cannot be ignored, especially in the case of common carotid artery variation. Additionally, carotid artery puncture approach requires strict preoperative cerebrovascular assessment such as CTA, while most patients in the real world do not undergo multi-modal imaging assessment within 6 hours. Strict preoperative assessment limits the application of this technology. Finally, there is no device specially designed for thrombectomy via direct carotid artery approach. Compared with conventional device via femoral artery approach, the length of this device should be shorter. We all use femoral artery access thrombectomy devices. A large part of the catheters in operation are in vitro, which is not accustomed to beginners.

## 6 Conclusion

In this study, we found significant differences in procedural or clinical outcomes in patients undergoing transcarotid versus transfemoral artery access for anterior circulation MT with unfavorable aortic arch and ICA anatomy. The main advantage of direct carotid artery access in thrombectomy is shortening the time to reperfusion. In well-selected patients, the transcarotid access may be a safer and more efficient approach for anterior circulation MT after LVO. However, further examination and technological developments are necessary.

## Author contributions

**Conceptualization:** Zhengzhou Yuan, Jinglun Li, Muke Zhou, Zuoxiao Li, Li He.

**Data curation:** Zhengzhou Yuan, Jinglun Li, Muke Zhou, Hua Luo, Zuoxiao Li.

**Formal analysis:** Zhengzhou Yuan, Jinglun Li, Hua Luo, Zuoxiao Li, Li He.

**Funding acquisition:** Li He.

**Investigation:** Zhengzhou Yuan, Jinglun Li, Muke Zhou.

**Methodology:** Zhengzhou Yuan, Jinglun Li, Muke Zhou, Hua Luo.

**Project administration:** Zhengzhou Yuan, Hua Luo.

**Resources:** Xiu Chen.

**Software:** Zhengzhou Yuan, Jinglun Li, Hongbo Zheng, Xiu Chen.

**Supervision:** Hongbo Zheng, Hua Luo, Xiu Chen.

**Validation:** Hongbo Zheng, Xiu Chen.

**Visualization:** Hongbo Zheng, Xiu Chen.

**Writing – original draft:** Zhengzhou Yuan, Li He.

**Writing – review & editing:** Zhengzhou Yuan, Zuoxiao Li.
